# The role of new vaccines in the prevention of otitis media

**DOI:** 10.1016/S1808-8694(15)30612-1

**Published:** 2015-10-18

**Authors:** Felippe Felix, Geraldo Augusto Gomes, Gustavo Augusto Porto Sereno Cabral, Jamerson Reis Cordeiro, Shiro Tomita

**Affiliations:** 1 Otorhinolaryngologist, master's degree student, Rio de Janeiro Federal University.; 2 Otorhinolaryngologist, master's degree student, Rio de Janeiro Federal University.; 3 Medical student, Rio de Janeiro Federal University.; 4 Medical student, Rio de Janeiro Federal University.; 5 Full professor of Otorhinolaryngology, Rio de Janeiro Federal University. Head of the Otorhinolaryngology Unit, Clementino Fraga Filho University Hospital, Rio de Janeiro Federal University. Rio de Janeiro Federal University.

**Keywords:** otitis media, pneumococci, vaccine, influenza virus

## Abstract

Otitis media is one of the most common infectious diseases of infancy; a reduction in its incidence would have a significant economic and social impact. Vaccines may play a role in the prevention of otitis media. This report discusses vaccines against pneumococci and influenza viruses. We reviewed the literature for results of studies examining the role of these vaccines in the prevention of otitis media. The 23-valent polysaccharide anti-pneumococcal vaccine did not modify the incidence of otitis media in children aged 2 years less, the age group with the highest incidence of otitis. The heptavalent anti-pneumococcal vaccine did not significantly reduce the incidence of otitis media overall. This vaccine did, however, reduce the number of episodes of otitis media with effusion and the number of recurrences; it also altered the profile of causative microorganisms by increasing otitis caused by different microorganisms. We found the inactivated anti-influenza virus vaccine to be effective in reducing otitis media during peak incidence periods of influenza. As these new vaccines are currently available in Brazil, otolaryngologists must be aware of their potential role and impact in the reduction of otitis media, to counsel patients appropriately.

## INTRODUCTION

Over 75% of children have at least one episode of acute otitis media (AOM) by age three years.^1^ It is estimated that 24.5 million cases of AOM occur annually in the USA, which are responsible for 33% of medical visits and 40% of antibiotic use in children aged below 5 years in that country.^2^ Thus, prevention of otitis media could have a significant economic and social impact.

Prevention methods include vaccines, which by definition are substances capable of activating an acquired immune response for defending organisms against an attack by viruses or bacteria.

In this review we will discuss the antipneumococcal and the inactivated influenza virus vaccines with the purpose of introducing these newly available vaccines to otorhinolaryngologists and showing their impact on the incidence of AOM.

### A) 23-valent pneumococcal polysaccharide vaccine:

This vaccine became available in 1983; it is composed of purified polysaccharides from the capsule of 23 pneumococcal serum types, as follows: 1, 2, 3, 4, 5, 6B, 7F, 8, 9N, 9V, 10A, 11A, 12F, 14, 15B, 17F, 18C, 19A, 19F, 20, 22F, 23F and 33F, which represent 85% to 90% of the serum types that are responsible for invasive disease in the USA, and a little over 80% in Brazil.^3^

Over age 2 years, the efficacy of this vaccine in preventing invasive pneumococcal disease is 56 to 81%. Efficacy has not been demonstrated - even in immunocompetent individuals - in the prevention of disease that course without bacteremia, such as otitis media.^4^

Notwithstanding its wide spectrum of action, the resulting immune response is T cell independent, that is, only B-lymphocytes are stimulated, which generates a short-lived response that does not induce immunological memory. Thus, an effective immune response is not generated in individuals aged below 2 years, particularly if otitis media is present.

It is a single-dose vaccine; revaccination is not routinely indicated. The indications are:

• Individuals aged over 65 years;
• Individuals aged between 2 and 65 years that are at an increased risk of severe pneumococcal infection and that are immunocompetent, but with chronic cardiovascular, pulmonary and hepatic diseases, diabetes, alcoholism, cerebrospinal fluid leak, and anatomical or functional asplenia (sickle cell disease);
• Individuals aged between 2 and 65 years that are at an increased risk of severe pneumococcal infection and that are immunodepressed due to human immunodeficiency virus infection, congenital immunodeficiency, leukemia, lymphoma, Hodgkin's disease, multiple myeloma, chronic renal failure or the nephrotic syndrome, following bone marrow or solid organ transplantation, or the use of immunosuppressant chemotherapy.

### B) Heptavalent pneumococcal conjugate vaccine:

The heptavalent vaccine was licensed in February 2000 in the USA, and is composed of the conjugated form of the CRM197 diphtheria protein (Prevnar in the USA, Prevenar in Europe) covering the following pneumococcal serum types: 4, 6B, 9V, 14, 18C, 19F and 23F.

The vaccination regimen is shown on Table 1, and is indicated for:

• All children aged below 2 years
• Children aged between 2 and 5 years and with a diagnosis of sickle cell anemia, anatomic or functional asplenia, chronic pulmonary or cardiac disease, diabetes, chronic renal failure / the nephrotic syndrome, cerebrospinal fluid leak and immunosuppressed patients;
• Children that will receive cochlear implants.

**Table 1:** Recommended heptavalent conjugate pneumococcal vaccine regimen in previously non-vaccinated infants and young children.

Age at 1st dose (months)	First series	Additional dose
2-6	3 doses at a 2-month interval	1 dose at 12-15 months
7-11	2 doses at a 2-month interval	1 dose at 12-15 months
12-23	2 doses at a 2-month interval	Unnecessary
24-59		
Healthy children	1 dose	Unnecessary
Children with underlying disease or immunosuppression	2 doses at a 2-month interval	Unnecessary

Conjugate vaccines consisting of pneumococcal capsule polysaccharides conjugated with a carrier protein (in this case the CRM197 diphtheria protein) induce T cell-dependent immunity, resulting in an increased antibody response and immunological memory; it is effective from birth, which is not the case of the 23-valent polysaccharide vaccine.

The efficacy of the conjugate vaccine against invasive pneumococcal disease and pneumonia in children has been demonstrated in many studies. There has been at least an 85% decrease in the incidence of invasive pneumococcal disease caused by the serum types present in vaccines, and a 20% decrease in pneumonia, which has been demonstrated radiographically.^5^, ^6^ What decrease might be expected in otitis media, considering that:

• *Streptococcus pneumonia* e causes about 50% of AOMs^7^;
• Vaccine serum types include about 75% of the causing agents of pneumococcal AOM^7^;
• The estimated efficacy of the heptavalent conjugate vaccine is 60%^5^, ^8^?

There would then be an expected 15 to 20% decrease in the general incidence of AOM. Various studies, however, have indicated that there has been about a 6% decrease.^8^, ^9^ A substitution phenomenon may explain this situation, whereby certain pneumococcal serum types and other bacteria not included in the vaccine may have proliferated and occupied the place of those that are included in the vaccine. There has been a 27 to 33% increase in pneumococcal AOMs caused by other serum types not included in the heptavalent vaccine.^6^, ^10^, ^11^ Additionally, according to Block et al.,^12^ there has been a proportional increase in AOMs caused by H. influenza and M. catarrhalis in post-vaccination periods (see Table 2) with a corresponding decrease in the incidence of pneumococcal AOMs.

**Table 2:** Distribution of bacteria in the etiology of AOM pre-and post-heptavalent conjugate pneumococcal vaccination.

Block et al.	Pre-vaccination period	Post-vaccination period
*S. pneumoniae*	49%	32%
*H. influenza*	39%	53%
*M. catarrhalis*	9%	12%

There are certain significantly prevalent serum types in Brazil (1 and 5) not common in the USA, which cause up to 20% of pneumococcal infections.^13^ Vaccine serum types compose about 63.5% of the number of pneumococcal infections in Brazil,^13^ different from the 75% found in North-American studies. The expectation for Brazil, therefore, is a decrease below 6% in the general incidence of AOM.

Pneumococcal antibiotic resistance to penicillin, to macrolides and to multiple drugs has been reported in five serum types: 6B, 9V, 14, 19F and 23F.^14^, ^15^ These serum types are included in the heptavalent vaccine, and a decrease in the number of infections caused by these microorganisms is associated with decreased pneumococcal antibiotic resistance.

Since the vaccinated children acted as the main bacterial carriers, decreased bacterial dissemination had a positive effect also on the non-vaccinated population. There was a decreased circulation of resistant germs, resulting in a decreased incidence of pneumococcal infection by penicillin-resistant pneumococci in individuals aged between 20 and 39 years (a 47% decrease) and in individuals aged over 65 years (a 37% decrease) when comparing the pre- and post-conjugate vaccine period.^14^

A North-American study5 showed a 20.1% decrease in otitis media with effusion (OME) requiring ventilation tubes. In similar conditions, a Finnish study showed a 39% decrease.^16^

The decrease in the incidence of recurring otitis media (four episodes within six months or more than five episodes within a year) in vaccinated groups was 11.9%;5 in cases where the infection rate was higher (5 episodes within six months or more than six episodes within a year) the decrease was 22.8%.^5^ According to Veenhoven et al.,^17^ There was no change in the incidence of recurrences in children already with recurring otitis media that received the heptavalent vaccine or the 23-valent polysaccharide vaccine compared with controls. This suggests that currently available vaccines appear not to be useful once recurrence is installed.

Based on these data, there will be a change in the profile of post-heptavalent conjugate vaccine AOM. This alteration may be a lower probability that cases caused by S. pneumoniae serum types found in vaccines will occur, resulting in milder cases and a decreased possibility of OME and recurrences. Furthermore, a lower incidence of penicillin-resistant S. pneumoniae is also expected, followed by an increased incidence of *H. influenza* and *M. catarrhalis* infections requiring more antibiotics against organisms that produce beta-lactamase.

### C) The inactivated influenza virus vaccine:

Twenty percent of children with upper airway infection by the influenza virus will progress to AOM, particularly below age 2 years.^18^

Inactivated influenza virus vaccination may only be used in children aged above 6 months, and should be revaccinated annually due to constant virus mutation. Children aged below 9 years that are vaccinated for the first time should receive a booster vaccine after four to six months, followed by annual revaccination.^3^

In 2003, the North-American Advisory Committee on Immunization Practices recommended introducing the inactivated influenza virus vaccine in the USA for children aged between 6 and 23 months, since this measure - according to various studies - reduced hospital admittance.^19^ In Brazil, the National Immunization Program recommends the influenza virus vaccine as part of the routine vaccination for individuals aged 60 years or above, and for the following risk groups:^3^

• HIV/AIDS;
• Solid organ and bone marrow transplantation;
• Donors of solid organs and bone marrow registered in organ donation programs;
• Congenital immunodeficiency, cancer or therapy-induced immunodeficiency;
• Healthcare professionals and caretakers of immunodepressed patients;
• Chronic cardiac and lung diseases, and asthma;
• Anatomical or functional asplenia;
• Diabetes;
• Cystic fibrosis;
• Trisomy;
• Cochlear implants;
• Incapacitating chronic neurological diseases;
• Chronic users of acetylsalicylic acid;
• Chronic nephropathy / nephrotic syndrome.

Clement et al.^20^ and Hekkinen et al.^21^ demonstrated a 32 to 36% decrease in the incidence of AOM during the period of maximum prevalence of influenza virus infection after using the inactivated influenza virus vaccine.

Hoberman et al.^22^ showed that there was no change in the frequency of AOM in children aged between 6 and 24 months. This study was hotly contested, for when it was undertaken, there was a generally lower incidence of influenza; furthermore, the study did not include children from nurseries (which are more predisposed to influenza infection), and its mean age was lower than comparable studies (mean 18 months).

Ozgur et al.^23^ showed that there was a 50.9% decrease in the incidence of AOM in a groups of children aged between 6 months and 5 years, compared to controls; this study also demonstrated a 26.7% decrease in the total number of cases of otitis media with effusion in a 6-month follow-up period.

## FINAL COMMENTS

The heptavalent pneumococcal vaccine, although not decreasing significantly the general incidence of AOM, alters the microbiological profile of this disease; there is a lower incidence of antibiotic-resistant pneumococcal serum types, a lower incidence of recurring otitis media and of otitis media with effusion. Additionally, a possible reduction in antibiotic use may be expected, as less virulent microorganisms will become a more frequent cause of AOM.

The currently available inactivated influenza virus vaccine in Brazil leads to a decreased incidence of seasonal AOM, during the period in which virus infection is higher. There was, however, no change in the final result during other periods and in children with no risk factors or influenza, compared to controls.
